# Recurrent Hematomas following a Revision Total Hip Arthroplasty in Acquired Coagulation Factor XIII Deficiency

**DOI:** 10.1155/2019/4038963

**Published:** 2019-07-18

**Authors:** Yoshinori Takashima, Shingo Hashimoto, Tomoyuki Kamenaga, Masanori Tsubosaka, Yuichi Kuroda, Kazuhiro Takeuchi, Koji Takayama, Shinya Hayashi, Ryosuke Kuroda, Tomoyuki Matsumoto

**Affiliations:** Department of Orthopaedic Surgery, Kobe University Graduate School of Medicine, Kobe, 7-5-1, Kusunoki-cho, Chuo-ku, Kobe 650-0017, Japan

## Abstract

Coagulation factor XIII (FXIII) is the final enzyme in the coagulation cascade and plays an important role in catalyzing the intermolecular cross-linking of fibrin polymers. FXIII deficiency is a rare disorder that presents with recurrent soft tissue bleeding. In this case report, we describe a patient with recurrent hematomas, following a revision total hip arthroplasty (THA). A 50-year-old female patient with no past history of bleeding and with a normal perioperative coagulation profile presented with recurrent hip joint hematomas. Her plasma FXIII activity showed a slight decrease (69%). Therefore, the patient was diagnosed with an acquired deficiency and was administered FXIII to correct it. The bleeding did not recur once the FXIII activity had returned to a normal level (76%). At 2 months after the second evacuation procedure, the patient was discharged from the hospital in an ambulatory state. There has been no recurrence of a hematoma since. We managed a rare case of acquired FXIII deficiency, which highlighted that a patient can present with an acquired bleeding disorder despite having a normal coagulation profile. An acquired FXIII deficiency should be suspected in patients with inexplicable, sudden-onset bleeding, as early diagnosis and treatment are important to prevent life-threatening complications.

## 1. Introduction

Coagulation factor XIII (FXIII) exists as a protransglutaminase (FXIII-A2B2) enzyme in the plasma. The enzyme is composed of two catalytic A subunits (FXIII-A2) and two noncatalytic B subunits (FXIII-B2), held together by noncovalent bonds [1, 2]. FXIII is the final enzyme in the coagulation cascade pathway and plays an important role in catalyzing the intermolecular cross-linking of fibrin polymers, thereby stabilizing the fibrin clot [3]. Patients with FXIII deficiency present with symptoms of recurrent soft tissue bleeding and delayed wound healing. To our knowledge, there are few published papers on the management of recurrent hip joint hematoma following a revision total hip arthroplasty (THA), in patients with FXIII deficiency. We report the case of a patient with recurrent postoperative hematomas that led to the diagnosis of this rare bleeding disorder.

## 2. Case Report

**Figure 1:**
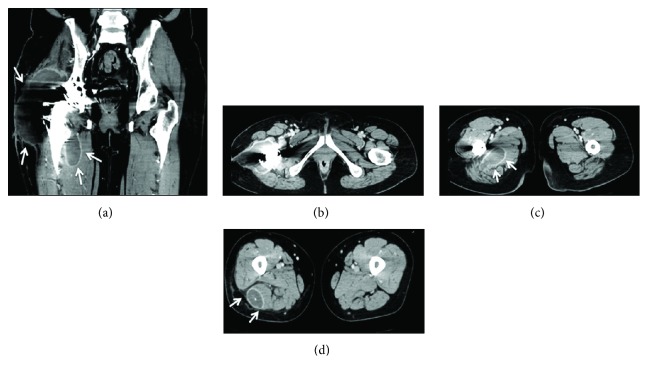
(a–d) Contrast computerized tomography images of the pelvic cavity at 10 days after the first evacuation procedure. A mass lesion was observed around the right hip joint. Although no active bleeding was observed, a hematoma was visible in this scan (white arrows).

**Figure 2:**
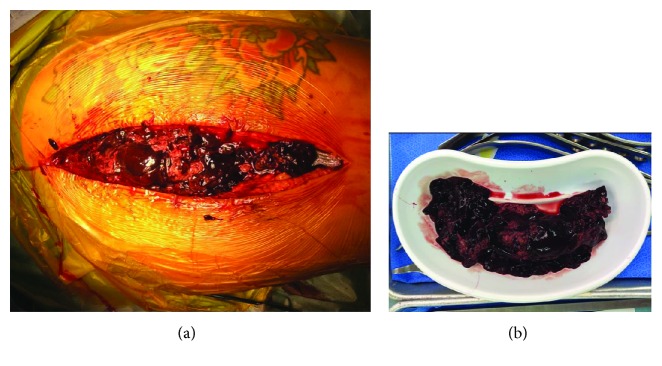
(a) Intraoperative finding—a large hematoma identified around the right hip. (b) The extracted hematoma, after the second evacuation procedure.

A 50-year-old woman underwent a THA of the right hip by the Dall approach, at another hospital, for an idiopathic osteonecrosis of the femoral head. However, she experienced frequent dislocation of the operated joint. She was then referred to our hospital the same year. The patient presented only with right-sided, lateral thigh pain. There was no past history of excessive bleeding following trauma, dental procedures, or surgery. She did not have a family history of excessive bleeding too. Her coagulation profile was within normal limits, with an activated partial thromboplastin time (APTT) of 29.8 s and a prothrombin time international normalized ratio (PT-INR) value of 0.98. A revision THA was performed using constrained liner, and no unexpected bleeding was observed during the operation. However, on postoperative day 21, the patient suddenly experienced acute pain and swelling of the right upper thigh. Her blood tests showed a C-reactive protein level of 495.23 nmol/L, a white blood cell count of 10.9 × 10^9^/L, and a hemoglobin value of 80 g/L. Her coagulation profile remained normal with APTT and PT-INR values of 28.2 s and 1.11, respectively. We suspected a postoperative infection or a hematoma and performed an emergency operation for an evacuation (of the hematoma), followed by irrigation of the operative site. Apart from a large hematoma identified at the operative site, there was no other significant finding. A tissue culture of the evacuated hematoma tissue did not yield any growth. At 10 days after the evacuation, the surgical wound site was found to be discharging a bloody exudate. A contrast computed tomography (CT) showed a mass lesion extending from the right hip to the proximal femur (Figure 1). The CT imaging and angiography did not reveal any active bleeding. At this time, her blood tests showed a further decrease in hemoglobin to 67 g/L, but her coagulation test results remained within normal limits with an APTT of 26.7 s and a PT-INR of 1.05 and a normal value of the von Willebrand factor. We then assessed the FXIII activity in the plasma, using a Berichrom FXIII kit (Dade Behring, Marburg, Germany) [4], which showed a slight decrease (69%). The patient was therefore diagnosed with an acquired FXIII deficiency, and a third procedure for an evacuation of the hematoma was performed (Figure 2), followed by an administration of human plasma-derived FXIII (Fibrogammin-P®; CSL Behring GmbH, Marburg, Germany) concentrate, at a dose of 1200 U/day for 5 days. After the treatment, the FXIII activity returned to normal levels (76%), and the bleeding did not recur. At 2 months after the second evacuation procedure, the patient was discharged from the hospital in an ambulatory state, mobilized with the aid of a T-cane. There has been no recurrence of a hematoma since.

## 3. Discussion

The most important inference derived from our experience in managing this patient was that an acquired FXIII deficiency should be unfailingly considered in the differential diagnoses when recurrent bleeding after THA is encountered, even when the coagulation profile remains within normal limits. Few studies have reported the complication of a hematoma around an operated hip joint as a result of FXIII deficiency.

The mechanism of FXIII deficiency is unknown and is associated with severe bleeding, spontaneous intracranial hemorrhages, and poor wound healing [2]. In the final step of the coagulation pathway, thrombin activates FXIII, which in turn triggers a cross-linkage of fibrin, a process essential for clot formation. FXIII strengthens fibrin thrombi and protects the fibrin clot from enzymolysis [5]. The resultant blood clot is thus stabilized by FXIII in the coagulation pathways. FXIII also protects against infection by immobilizing and destroying bacteria, in addition to promoting phagocytosis by macrophages [6]. FXIII deficiency is classified as either a congenital or an acquired illness. Hereditary FXIII deficiency is a rare bleeding disorder (incidence of 1 in 5 million) [7]. The major cause is a genetic defect in FXIII. An acquired FXIII deficiency may arise because of specific autoantibodies but may also be consequent to other underlying diseases that result in a decreased synthesis or an increased consumption of FXIII. A decrease in FXIII in adults has been attributed to a variety of causes such as chronic liver disease, leukemia, Henoch-Schönlein purpura, autoimmune diseases, drugs, and trauma [8]. Also, Kanda et al. [9] reported that an infection of the prosthesis subsequent to a revision THA might be one of the risk factors.

Postoperative hematomas tend to occur in patients who have received anticoagulation or hormonal therapy or in those who have thrombocytopenia and hemorrhage. Almost all hematomas spontaneously reduce through internal absorption, but a huge hematoma that may occur following hip surgery can cause nerve palsy, acute thigh pain, and swelling [10, 11]. A hematoma is also a risk factor for a periprosthetic infection [12]. Our patient had no history of risk factors, and her coagulation profile (PT, APTT, platelet count, platelet function assay, and bleeding time) remained within a normal range. However, she developed recurrent hematomas at the operative site, following the revision arthroplasty. Our differential diagnoses for this patient also included other hemorrhagic disorders, such as von Willebrand disease, *α*_2_-antiplasmin deficiency, platelet factor 3 deficiency, dysfibrinogenemia, and plasminogen activator inhibitor-1 deficiency [3]. In our case, there was a minor decrease in FXIII activity (69%). However, a FXIII activity level at which hemorrhage may occur is variable and patient-specific [13, 14]. The localized soft tissue damage following the revision THA and the minor FXIII deficiency may have acted additively to affect the bleeding. In addition to that, after 5 days of supplementation, the FXIII activity returned only to 76%. This almost unchanged FXIII activity after the treatment is a strong indicator that a relevant consumption of FXIII has been taking place.

As the presence of a FXIII inhibitor could not be demonstrated conclusively in our patient, we considered her deficiency to have been caused by an increased consumption process. When an autoimmune inhibitor of FXIII is not detected in the plasma, the administration of FXIII concentrate is an effective treatment [15]. If FXIII is not available at hand, fresh frozen plasma administration is also acceptable. Conversely, if a FXIII inhibitor is detected in the plasma, additional immunosuppressive therapy using adrenocorticosteroids, cyclophosphamide, plasma exchange therapy, adsorptive therapy, or high-dose immunoglobulins would be required for an effective outcome [3, 15]. In our patient, the FXIII replenishment was remarkably effective. Additional therapy was not needed, and the patient made steady progress to recovery.

## 4. Conclusion

We managed a rare case of FXIII deficiency and conclude that a patient presenting with an acquired bleeding disorder can have a normal coagulation profile. An acquired FXIII deficiency should be a part of the differential diagnoses when sudden bleeding tendency is observed, especially in a postoperative patient. An early diagnosis and treatment are imperative in preventing the life-threatening complications of an acquired FXIII deficiency.
